# Esophagus Segmentation in CT Images via Spatial Attention Network and STAPLE Algorithm

**DOI:** 10.3390/s21134556

**Published:** 2021-07-02

**Authors:** Minh-Trieu Tran, Soo-Hyung Kim, Hyung-Jeong Yang, Guee-Sang Lee, In-Jae Oh, Sae-Ryung Kang

**Affiliations:** 1 Department of Artificial Intelligence Convergence, Chonnam National University, 77 Yongbong-ro, Gwangju 500757, Korea; tmtvaa@gmail.com (M.-T.T.); shkim@jnu.ac.kr (S.-H.K.); hjyang@jnu.ac.kr (H.-J.Y.); 2 Department of Internal Medicine, Chonnam National University Medical School and Hwasun Hospital, Hwasun 58128, Korea; droij@jnu.ac.kr; 3 Department of Nuclear Medicine, Chonnam National University Medical School and Hwasun Hospital, Hwasun 58128, Korea; campanella9@naver.com

**Keywords:** esophagus segmentation, deep learning, spatial attention module

## Abstract

One essential step in radiotherapy treatment planning is the organ at risk of segmentation in Computed Tomography (CT). Many recent studies have focused on several organs such as the lung, heart, esophagus, trachea, liver, aorta, kidney, and prostate. However, among the above organs, the esophagus is one of the most difficult organs to segment because of its small size, ambiguous boundary, and very low contrast in CT images. To address these challenges, we propose a fully automated framework for the esophagus segmentation from CT images. The proposed method is based on the processing of slice images from the original three-dimensional (3D) image so that our method does not require large computational resources. We employ the spatial attention mechanism with the atrous spatial pyramid pooling module to locate the esophagus effectively, which enhances the segmentation performance. To optimize our model, we use group normalization because the computation is independent of batch sizes, and its performance is stable. We also used the simultaneous truth and performance level estimation (STAPLE) algorithm to reach robust results for segmentation. Firstly, our model was trained by k-fold cross-validation. And then, the candidate labels generated by each fold were combined by using the STAPLE algorithm. And as a result, Dice and Hausdorff Distance scores have an improvement when applying this algorithm to our segmentation results. Our method was evaluated on SegTHOR and StructSeg 2019 datasets, and the experiment shows that our method outperforms the state-of-the-art methods in esophagus segmentation. Our approach shows a promising result in esophagus segmentation, which is still challenging in medical analyses.

## 1. Introduction

Cancer is not only one of the critical worldwide public health problems but also a leading reason for millions of deaths every year. Nowadays, cancer types are becoming much more popular with the rapidly increasing number of patients. The treatment is split into multiple stages, where radiotherapy treatment is one of the essential steps. During radiotherapy, the organs near the tumor can be damaged, which are called organs-at-risk (OARs). Thus, protecting the OARs raises an important concern. One way to avoid OARs injury is segmentation of OARs from CT images in the treatment planning [1,2]. Therefore, OARs segmentation remains an active area of research. In previous clinical practices, OARs segmentation can be done manually by doctors and radiologists. However, this is time-consuming because of considerable slices in CT scans and the requirement of high accuracy. For those reasons, nowadays, the automated segmentation system is receiving more interest from researchers.

The esophagus is very difficult to segment in CT scans [3], and it is one of the most important OARs in radiotherapy due to its radiosensitive mucosa, so precise segmentation is indispensable. The boundary between the esophagus and the other organs is often unclear. Because of the low contrast and small size of the esophagus in the human body, it is hard for doctors or oncologists to accurately locate the position of the esophagus in the CT images. It is complicated to distinguish the esophagus on medical images, even for specialists. The proposed method aims to support the specialist or the medical doctors in radiotherapy treatment planning. In recent years there has been quite a lot of research focusing on the field of esophagus segmentation [4,5,6,7,8]. However, most of these methods ignore the spatial information from organs. Additionally, they usually evaluate only one dataset. We proposed a method based on the combination of the deep learning approach and conventional algorithm. The model is built from a variant of U-Net structure, attention mechanism, and atrous spatial pyramid pooling module [9] to capture the larger receptive field. Thus, our method can learn spatial information and focus on the meaningful area better than other methods. Several studies employ group normalization [10] (GN) to optimize their segmentation models [11,12,13]. A biomedical semantic segmentation model [11] shows that when they used GN, they achieved higher accuracy results than using other kinds of normalization. Also, a lightweight deep convolutional neural network [12] using GN shows that this model has high performance in the biomedical image segmentation field. Recently, an end-to-end segmentation network [13] called Dilated Multi-ResUNet also presents the effectiveness of GN in reducing the adverse impact brought by the small batch size. From the advantages of GN in the segmentation area, we employ it in our approach to optimize the model.

In summary, the contributions of this work can be summarized as:

1. We proposed an automated framework for segmentation of esophagus with high accuracy. The segmentation framework also can be applied to other types of organs. The ablation study showed that it achieved competitive results compared to the state-of-the-art ones.
2. The proposed model takes advantage of the spatial information from the attention module. With a larger receptive field from the atrous spatial pyramid pooling module, the feature of the esophagus is better captured. Also, we employ GN in our model to get high performance and stable results.
3. We construct the segmented image into two-dimensional (2D) and 3D images. Thus, they can assist doctors or specialists better than only shown in one kind of 2D or 3D.
4. Experimental results from two public datasets SegTHOR and StructSeg, demonstrate our results in segmentation of esophagus outperformed the state-of-the-art methods.

The rest of the paper is organized as follows. Section 2 introduces the related works addressing the thoracic organs at risk segmentation and esophagus segmentation. The details of our method, such as the spatial attention module, our segmentation model, and the post-processing step, are presented in Section 3. The achieved experimental results of our approach in SegTHOR and StructSeg datasets are reported in Section 4. Finally, the conclusions section is given in Section 5.

## 2. Related Works

### 2.1. Thoracic Organs at Risk Segmentation

To human perception, several earlier systems were developed on the traditional approaches, including techniques such as atlas-based and statistical-based methods [14,15,16,17,18,19,20,21]. The methods mentioned above have been holding the leading role for a long time. However, they are not without limitations, one of which is the requirement of hand-crafted features, and the other is that complicated cases cannot be handled effectively. Nowadays, deep learning is well-known as a superb way to solve these problems. It shows exceedingly outstanding accomplishment in many tasks such as classification, object detection, and especially segmentation. This framework demonstrates an end-to-end learning approach. Instead of making hand-crafted features via convolution, pooling layers, and activation functions, the network can extract the features from which patterns are recognized via the backward propagation of errors process. Deep convolutional neural networks (DCNN) are widely applied in segmentation models and achieved significant accomplishments in various fields. The medical research area also had large benefits from DCNN.

Segmentation can be considered an extension of classification where the network predicts the category (organs, background, etc.) of each pixel of the input image. A fully Convolutional Network (FCN) was first introduced by Long et al. [22] for semantic segmentation. In this network, there is a slight difference compared to the classification network. More formally, the last fully connected layers are removed and replaced by fully convolution layers. The higher resolution feature maps are concatenated with upsampled lower resolution and then passed to the next fully convolution layers to achieve better accuracy. FCN is widely used for multi-organ segmentation by various approaches: 2D [23,24], 2.5D [25] and 3D [26,27].

In 2015, Ronneberger et al. proposed U-Net [28] built upon the idea of FCN and using the concepts of deconvolution introduced by [29]. The contracting path, well known as the encoder path, consists of multiple stages to extract the contexture of the object. In the expansion path, well known as the decoder path, the feature map from the contracting path is upsampled to match the feature size from previous decoders and concatenated together. This technique enables the network to capture both context and location information of the object.

Several novel architectures have appeared to show significant success in medical segmentation [30,31]. A framework employs 3D-U-Net to detect vascular boundaries [32]. The pix2pix model [33] is suitable for image-to-image translation tasks, where an input image is adjusted and generates a corresponding output image. The method is valuable at synthesizing images from ground truth, colorizing images, image segmentation. This framework shows a promising result for many image-to-image translation challenges, especially highly structured graphical outputs. A 3D framework called V-Net [34] is proposed for 3D image segmentation. This network trained end-to-end on MRI volumes reproducing prostate, and this net learns to predict segmentation for the whole volume at once. The approach directly uses 3D convolutions instead of employing the input volumes in a 2D slice-by-slice. Besides, a practical loss function explicitly designed for medical image segmentation is utilized for the training phase.

The SegNet [35] framework represents a deep convolutional neural network architecture for semantic pixel-wise segmentation. This network consists of an encoder path, a corresponding decoder path followed by a pixel-wise classification layer. The critical point of SegNet is the decoder path which includes a hierarchy of decoders corresponding to each encoder. A network architecture is based on employing dilated convolutions [36] to capture features at multi-scale images and densely connecting all feature maps. This framework can achieve accurate results while the model is easier to implement, train, and apply in practice and automatically adapts to different problems.

A novel Convolutional Neural Network (CNN), called USE-Net [37], integrated Squeeze-and-Excitation blocks [38] into U-Net to exploit adaptive channel-wise feature recalibration to boost the generalization performance. This framework achieves accurate prostate zonal segmentation results when trained on multi-institutional datasets. The approach is a valuable solution in medical imaging applications related to multi-institutional settings. Another deep learning framework for segmentation is presented by Rundo et al. [39] to automatically delineate the Central Gland and Peripheral Zone in the prostate gland. This study evaluates the generalization ability of CNN on two multi-centric MRI prostate datasets. The critical point in this study is that significant performance improvement through fine-tuning may require a massive dataset for pretraining.

A framework based on U-Net, with skip connections between contracting and expanding paths, is used for OARs segmentation [40]. In this model, the pixel shuffle is employed during the decoder as an upsampling operator. A novel multitask framework is proposed for OARs segmentation [41]. This framework includes a coarse segmentation network used to obtain the regions of interest (ROIs) localization. After that, multi-level ROIs are cropped from the encoder part to form a decoder for detail-preserving segmentation. Additionally, a deep learning framework for OARs segmentation in CT images is introduced in [42]. This method is based on a two-stage approach for the segmentation task. A 3D U-shape network is employed to get the localization of four organs at first. Then the output result is put into the same network again to achieve better segmentation results.

An approach that employs dilated convolutions and aggregated residual connections in the bottleneck of the U-Net variant is used to segment the OARs [43]. The model utilizes global context and dense information necessary to recognize boundaries between adjacent organs effectively. A 3D Enhanced Multi-scale Network (EMSN) [44] is proposed to segment the OARs. This framework is based on a variant of the 3D FCN network. The method uses a concatenation between preliminary prediction maps with the CT images to refine the prediction maps. Besides, this network adopts 3D dilated convolution and residual connections to enlarge the receptive field kernel of convolution without loss of resolution and avoids gradient degradation during back-propagation, respectively.

Another multitask framework for OARs segmentation is proposed in [45]. In this model, there are two tasks which are the main and the auxiliary tasks. In the main task, the model tries to segment OARs, while the auxiliary task is the multi-label classification of organs in CT images. A new loss function called weighted mean Cross-Entropy loss function is introduced to optimize the learning process during training the model. A multi-resolution 3D V-Net network is presented in [46] to segment thoracic OARs in CT images. The model employs two resolutions from images for the learning process. A variant of the V-Net model called VB-Net is proposed for training both resolutions. In the coarse resolution case, the model can robustly localize the organs, while the fine resolution can help accurately refine each boundary of the organ.

Additionally, a two-stage network for multiple organs at risk in the head and neck area segmentation is proposed in [47]. In the first stage, a coarse network on size-reduced medical images was employed to find the organs of interest. After that, a fine network for segmentation on full-resolution images was applied to get the final segmented maps. The approach shows good performance for the segmentation of structures in the neck and head area.

Besides the deep learning approaches, several methods regarding OARs segmentation are related to machine learning [48,49]. A semi-automatic method for Epicardial Fat Volume (EFV) segmentation and quantification is proposed by [48]. The key point in this approach is that it does not require any initial training or modeling phase to set up the system, unlike other supervised machine learning approaches. The EFV quantification and analysis method is a valuable tool to assist experts in diagnosis and therapy. Also, a method for heart segmentation based on watershed and active contour models was proposed in [49]. In this approach, at the first stage, the bilateral filtering technique is used to reduce the noise of the cardiac CT images. In the next stage, initial seed contours are determined by the watershed segmentation method. Finally, precise segmentation boundaries for whole heart CT images are obtained by the active contour model.

Although these approaches demonstrate potential in medical segmentation, the 3D model consumes colossal computational resources. Also, most of the methods do not focus on spatial information of the organs. Therefore, building an efficient 2D model with spatial attention is an attractive way for researchers.

### 2.2. Esophagus Segmentation

Several studies focus on solving the problem of esophagus segmentation [4,5,6,7,8]. A model FCN [4] is used for segmentation of esophagus. This model employs low-level features with high-level information, effectively combining local and global information to improve segmentation accuracy. A method based on a 2D model called U-Net Plus [5] is proposed to segment the esophagus from the 2D CT slice. This architecture enhances the feature extraction performance of complex abstract information thanks to two special blocks. According to the authors, their method is evaluated on a database that contains 15 CT images totalizing more than two thousand slices. Their results achieved an average Dice value of 0.79. Although the performance of this method looks good, they used a small size dataset of only 15 exams.

The Channel Attention mechanism is employed inside the method [6] to distinguish the esophagus and surrounding area by emphasizing and inhibiting channel features. This method integrated a Channel Attention Module (CAM) and Cross-level Feature Fusion Module (CFFM) into a deep learning model to strengthen the generalization ability of the network by employing high-level features to low-level features. An atlas-based deep learning approach [7] is used to segment the esophagus. This method includes five main steps proposed for esophagus segmentation for better planning of radiotherapy in CT. These steps are image acquisition, volumes of interest segmentation, preprocessing, esophagus segmentation, and segmentation refinement.

U-Net neural network combined with several variations of backbones [8] is proposed for esophagus segmentation. This is a semi-automatic labeling method with detection and execution components to solve the labeling challenge. The detection phase aims to identify the category to which each slice belongs. Several backbones are employed as the encoder of the U-Net network to extract features. The difficulties in esophagus segmentation, even by a specialist, take so much time and is susceptible to human error [3]. This framework employs a CNN and an active contour model (ACM). The outputs from CNN and ACM are applied to a random walker algorithm. According to the authors, this method is evaluated on a dataset of 50 patients. Their Dice coefficient result achieves 0.76.

A probabilistic method for segmentation of esophagus [50] is proposed to segment the esophagus automatically. They detect the ROI first by finding salient anatomical landmarks. After that, prior knowledge about the esophagus region is used to infer the approximate boundary of the esophagus by finding the largest value of the posterior estimate. Two different ways of describing and inferring form information are contrasted: A “detect and connect” method which uses a combination of the Markov chain model and a particle filter. Finally, the non-rigid surface that emerges from this approach is deformed to better conform to the limitations of the organ. A skeleton-shape model to guide the segmentation [51] is proposed to segment in thoracic CT scans of the 3D esophagus. Although the method is automatic, it depends on generating a skeleton model based on the specialist marking.

Again, the difficulty in esophagus segmentation is presented by Trullo et al. [4]. This paper proposed a fully automatic method consisting of only two steps. The first step is that a convolutional neural network estimates the location of the esophagus. And then, the area calculated in the previous step is cropped and put into the same network. This method needs a crop in the esophagus location. Although this is the automated method, if the first network ignores the position of the esophagus or marks it in the wrong area, the second network meets the problem of segmenting it. A deep learning approach for various organ segmentation is introduced in [52]. In this method, the au-thors crop the area of each organ based on its previous location. Finally, the segmented results of each organ are joined to create the final multi-organ segmentation. This framework is evaluated on a dataset that consists of 36 CT images, and it has an average Dice value of 0.72 in segmentation of esophagus.

As can be seen in the literature, most studies show two problems when they research esophagus segmentation. Firstly, the esophagus is very difficult to segment, even for doctors and specialists. The difference between the contrast of the esophagus boundary with the other organs in most slices is usually unclear. Secondly, the works often find the position of the esophagus based either on probabilistic models, at atlases, or other locations of the organs to decrease the region of interest. Therefore, the final segmented result usually depends on the previous preprocessing steps.

In this paper, we propose a variant of the U-Net network for esophagus segmentation. This model can also be applied in other organ segmentation. The network leverages pre-trained models from ImageNet to extract precise context features. We design a decoder with the spatial attention module to refine the object location accurately in the expansion path. We also employ the STAPLE [53] algorithm to boost the final performance. The experiments show the effectiveness and robustness of our network by achieving high results on esophagus segmentation on StructSeg2019 and SegTHOR datasets.

## 3. Materials and Methods

In this section, we firstly review the concepts of spatial attention. After that, the proposed architecture for segmentation of esophagus is presented.

### 3.1. Spatial Attention Module

**Figure 1:**
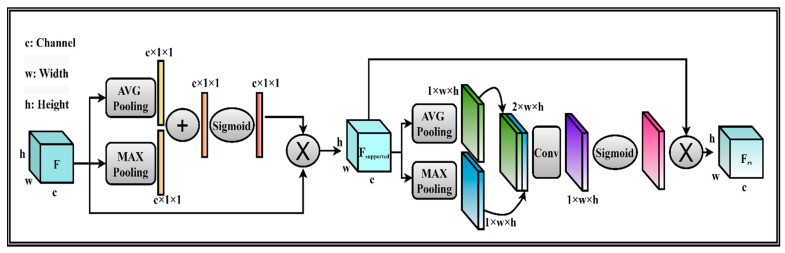
The proposed spatial attention module.

To human perception, attention holds an important role [54,55,56]. It is a fact that humans are often impressed by salient parts from the whole scene and then focus on them to capture and understand visual structure. Inspired by this concept, there were many attempts in trying to bring attention to deep learning networks. Most of them share the same approach. In this paper, a spatial attention module was employed to utilize the spatial relationship of features. The spatial attention answers the question “where” valuable parts are. Firstly, the average pooling and max pooling operations are applied along the channel axis, and then the outputs are concatenated to create a useful feature. The highlighting informative regions are exploited by using pooling operations along the channel axis [57]. The concatenated feature above is fed into a convolution layer to create the spatial attention map representing emphasized or suppressed areas. Given the feature map F, the attention mechanism is to procedure the attention map A(F), which indicates the most important features. We also used the combination of max pooling and averaged pooling S to summarize feature information. It can be supported by the original feature information. Refined feature map $F_{refined}$ is computed as: $F_{refined}=F⨂A\left(F\right)$ where ⨂ denotes element-wise multiplication. We employed a spatial attention module (SAM), which is called $A_{s}$. It is used for exploiting inter-spatial relationships. The attention operation is described as follows:

(1) $$F_{supported}=F⨂S\left(F\right)$$

(2) $$F_{rs}=F_{supported}⨂A_{s}\left(F_{supported}\right)$$

where $F\in \;{\mathbb{R}}^{C\times H\times W}\;$, $S\left(F\right)\in \;{\mathbb{R}}^{C\times 1\times 1},\;A_{s}\left(F_{supported}\right)\in \;{\mathbb{R}}^{1\times H\times W}\;$. H,W,C are height, width, and the number of channels of feature map F. The supported features, refined spatial features after applying pooling and spatial attention module denoted by S and $F_{rs}$, respectively. The details of the spatial attention module show in Figure 1.

### 3.2. The Proposed Method

**Figure 2:**
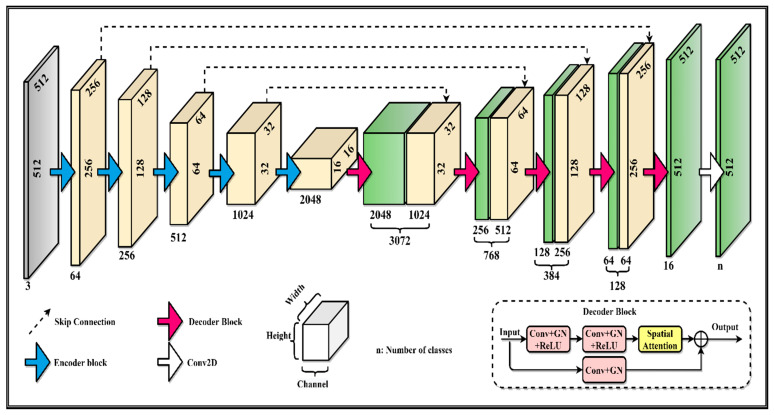
Details of our proposed model.

We present the proposed method based on a model with spatial attention, as in Figure 2. With simple problems, a single label for training is enough for a good result. However, multiple labels are better for training for complex problems than a single label [58]. The esophagus is one of the most challenging organs to segment because of its small size, ambiguous boundary, and very low contrast in CT images. Thanks to the multi-label strategy, meaningful spatial relationships are utilized for distinguishing the esophagus from others. U-Net has the capability to train with a small dataset. Especially, the medical dataset is relatively rare and small due to the fact that not so many datasets were published or available to the public. In addition, it is clear that the popular models require a large-scale dataset (up to million images) to be generalized and avoid overfitting. Therefore, it is not a good idea to train a typical U-Net from scratch with initialized random weights. It is well known that the pre-trained models on ImageNet are widely used for transfer learning and achieve significant success on many tasks. Thus, we use pre-trained models (Resnet34 [59] and SEResNext50 [38]) as the encoder. For the ResNet family, there are major blocks. Each block consists of several convolutions, pooling layers, and activation functions. The first block is called stem layers, including 2D convolution, GN [10], and ReLU. The rest of the four blocks share a similar structure, including bottleneck and basic blocks. Note that GN divides the channels into groups and calculates the variance and mean for normalization within each group. Therefore, the computation is independent of batch sizes, and its performance is stable in a wide range of batch sizes. We take each block corresponding to the encoder stage in the same sequence as the original model. The output feature map is not only used for feeding the next block but also for skip connection. We denote $\Phi_{i_{p}}^{k},i=\left\{0,1,2,3,4\right\}$ as the encoder operation. The encoder Φi takes a feature map having k channels as the input then produces the output feature map having p channels. Similarly, $\Omega_{i_{p}}^{k},i=\left\{0,1,2,3,4\right\}$ denotes for the decoder operation. The encoder is described as following:

(3) $$\Phi_{0_{m_{0}}}^{n_{0}}\to \Phi_{1_{m_{1}}}^{m_{0}}\to \Phi_{2_{m_{2}}}^{m_{1}}\to \Phi_{3_{m_{3}}}^{m_{2}}\to \Phi_{4_{m_{4}}}^{m_{3}}\;$$

where $n_{0}=3,$ which is the number of channels of CT slice images. The feature map channels of encoders: $(m_{0},m_{1},\;m_{2},m_{3},m_{4})\;$are (64, 64, 128, 256, 512) for Resnet34 and (64, 256, 512, 1024, 2048) for SEResNext50.

The decoder operations are:

(4) $$\Omega_{0_{m{’}_{0}}}^{m_{4}+m_{3}}\to \Omega_{1_{m{’}_{1}}}^{m{’}_{0}+m_{2}}\to \Omega_{2_{m{’}_{2}}}^{m{’}_{1}+m_{1}}\to \Omega_{3_{m{’}_{3}}}^{m{’}_{2}+m_{0}}\to \Omega_{4_{m{’}_{4}}}^{m{’}_{3}}\;$$

The feature map channels of decoders: $(m{’}_{0},m{’}_{1},\;m{’}_{2},m{’}_{3},m{’}_{4})\;$are (256, 128, 64, 32, 16) for both of backbones. The decoder consists of two branches. The first branch includes 2 × [Conv → Groupnorm → ReLu]. Each convolution layer has kernel size 3 × 3, padding and stride are 1 × 1. We employ spatial attention modules following convolution blocks to refine the feature map after that. The second branch has only one block [Conv → Groupnorm] with 1 × 1 convolution to reduce the dimension of the feature map. This branch is inspired by the Inception [60] and downsampling of ResNet [59] architecture. Finally, two feature maps produced by two branches are merged by element-wise addition. Figure 2 shows our proposed model.

### 3.3. Post Processing Step with STAPLE Algorithm

**Figure 3:**
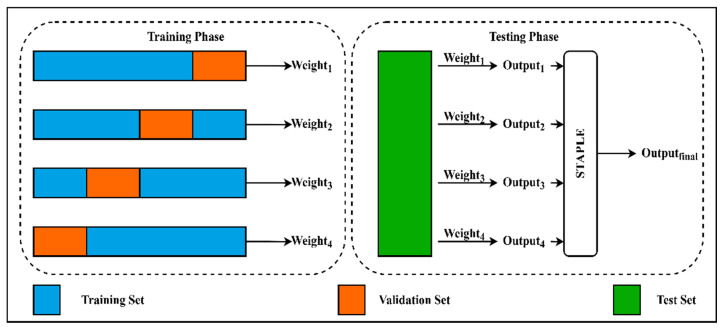
The overall architecture of our method.

**Figure 4:**
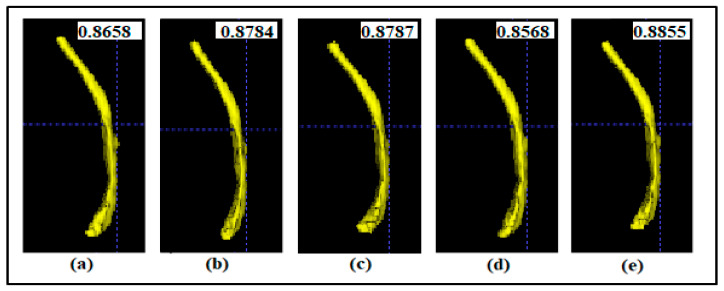
3D visualization of esophagus segmentation results from patient 49th in the test set of SegTHOR dataset. The number indicates the Dice score, which was obtained from the organizer of the challenge. (**a**) Result from the weight of fold-1-trained; (**b**) Result from the weight of fold-2-trained. (**c**) Result from the weight of fold-3-trained. (**d**) Result from the weight of fold-4-trained. (**e**) Result from our method.

A deep learning-based method for fully automatic segmentation of multiple closely spaced brachytherapy catheters in intraoperative MRI presented in [61] is used in the post-processing step to robust their segmented results. In the training phase, the model was trained using 5-fold cross-validation. Then, the candidate labels generated by each fold were fused by using a majority voting algorithm. However, the STAPLE algorithm proved better than major voting [62], and an improvement of the accuracy was also observed when applying the STAPLE algorithm to automatic segmentation results [63]. Thus, our approach employed STAPLE to robust the segmented results. STAPLE is an expectation-maximization algorithm for simultaneous truth and performance level estimation. This method considers a collection of segmentations and calculates a probabilistic estimate of the true segmentation and a measure of the performance level represented by each segmentation. The probabilistic estimate of the final output segmentation is formed by estimating an optimal combination of the segmentations, weighting each segmentation result depending upon the estimated performance level, and employing a prior model for the spatial distribution of structures being segmented as well as spatial homogeneity constraints. In the SegTHOR dataset, we employ 4-fold cross-validation. That means we have four checkpoints from the four times training model. While the typical approach uses the mean or max operator based on the output from four times testing, this paper uses the STAPLE algorithm to combine four results from our four checkpoints. We convert each segmented image of multiple organs into multiple segmented images for each separate organ to apply the STAPLE algorithm. We then used the STAPLE technique for different results of each weight from k-fold training on each organ. After that, we get results of multiple 2-class segmented maps for each kind of organ. Finally, we refactor these 2-class segmented maps into a completely segmented image with multiple segmented organs in one image. The experiments show the effectiveness and robustness of our network combined with the STAPLE algorithm by achieving high results on esophagus segmentation on the SegTHOR dataset. We present the overall architecture of our method in Figure 3. ${\mathrm{Weight}\;}_{1}$ is the weight from training in fold 1. The ${\mathrm{Weight}\;}_{2}$, ${\mathrm{Weight}\;}_{3}$ and ${\mathrm{Weight}\;}_{4}$ are similar. ${\mathrm{Output}\;}_{1}$ is the segmented map result when we use ${\mathrm{Weight}\;}_{1}$ for the predicted result. The ${\mathrm{Output}\;}_{2}$, ${\mathrm{Output}\;}_{3}$ and ${\mathrm{Output}\;}_{4}$ are similar. The ${\mathrm{Output}\;}_{\mathrm{final}}$ is the segmented result which gets from combining four segmented maps from four weights by STAPLE algorithm. Figure 4 shows a 3D visualization of esophagus segmentation results from patient 49th in the test set of the SegTHOR dataset.

## 4. Experimental Results

### 4.1. Dataset

We used StrucSeg 2019 and SegTHOR datasets to evaluate the performance of our proposed network. All images are 3D CT scans. We extract all the slices from 3D volume to 2D images in each patient. The Houndsfield Unit (HU) values are normalized into the range of [0,1] as follows:

(5) $$I=\frac{I-A}{B-A}\;$$

where B; A are the upper and lower boundary of HU. We select B = 400, A = −1000 in our experiments on StructSeg 2019 dataset. With the SegTHOR dataset, we choose *B* = 400, *A* = −400 in our experiments.

#### 4.1.1. StructSeg 2019 Dataset

**Figure 5:**
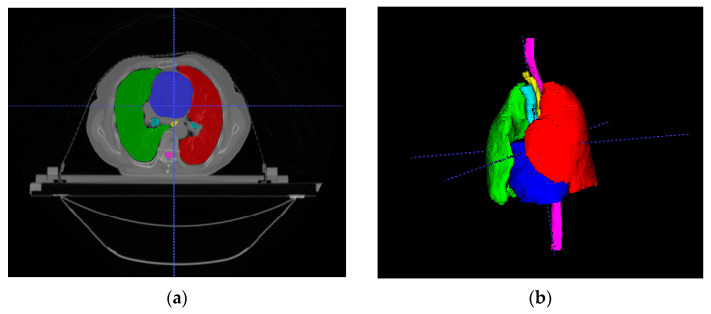
Example of OARs from StructSeg 2019 dataset. (**a**) 2D image; (**b**) 3D image. Each OAR is shown in a different color. The green region is the left lung; the red region is the right lung; the pink region is the spinal cord; the turquoise is the trachea; the blue region is the heart, and the yellow region is the esophagus.

We used the StrugSeg dataset to evaluate the performance of our proposed network. The dataset has CT scans of 50 lung cancer patients for training and ten patients for testing. Each scan is annotated by one expert and verified by another one. There are six annotated OARs: left lung, right lung, spinal cord, esophagus, heart, and trachea. We split the 50 3D images into file groups for 5-fold cross-validation. Image intensity values were truncated from −1000 to 400 HU to omit irrelevant information. The example of OARs from the StructSeg 2019 dataset is shown in Figure 5. The green region is the left lung, the red region is the right lung, the pink region is the spinal cord, the turquoise is the trachea, the blue region is the heart, and the yellow region is the esophagus.

#### 4.1.2. SegTHOR Dataset

**Figure 6:**
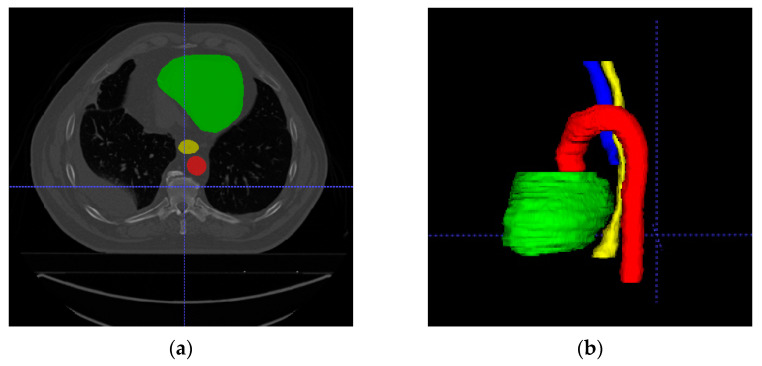
Example of OARs from SegTHOR ISBI 2019 dataset. (**a**) 2D image; (**b**) 3D image. Each OAR is shown in a different color. The green region is the heart; the yellow region is the esophagus; the blue region is the trachea; the red region is the aorta.

This dataset comes from the ISBI 2019 SegTHOR challenge. There are 40 labeled scans with four thoracic organs, including the esophagus, heart, trachea, and aorta, in the dataset. We split the 40 CT images into four groups for applying 4-fold cross-validation. Image intensity values were truncated from −400 to 400 HU to omit irrelevant information. The example of OARs from the SegTHOR 2019 dataset is shown in Figure 6. The green region is the heart, the red region is the esophagus, the blue region is the trachea, and the yellow region is the aorta.

### 4.2. Evaluation Metrics

We use the Dice score for measuring the overlapped volume ratio between predicted segmentation map *T* and ground truth *N*.

(6) $$DSC\left(T,N\right)=\frac{2\times \left|T\cap^{\;}N\right|}{\left|T\right|+\left|N\right|}$$

Hausdorff Distance (HD) is the maximum distance between the boundary of predicted segmentation *T* and ground truth *N,* which is defined as:

(7) HD(T,N)=max{dist(T,N),dist(N,T)} 

where

(8) $$dist\left(T,N\right)=\underset{x\in T\;}{sup}\;\underset{y\in N\;}{inf}\;d\left(x,y\right)$$

and

(9) $$dist\left(N,T\right)=\underset{x\in N\;}{sup}\;\underset{y\in T\;}{inf}\;d\left(x,y\right)$$

sup and inf are supremum and infimum of each set correspondingly. 95% HD (HD95) based on calculating the 95th percentile of the distances between *T* and *N*. This evaluation metric eliminates the impact of the outliers. In this paper, the value of HD is computed in millimeters (mm).

### 4.3. Training Model

#### 4.3.1. With StructSeg Dataset

We address the problem as seven classes segmentation task. There are six OARs and a background. Because of one pixel for one class, we select the Softmax function to handle this task. Our method is evaluated in the Dice and HD metrics. We employ a combination of Dice loss and Cross-Entropy loss as follows:

(10) $$Lcombination=\theta \times Diceloss\left(\hat{t},t\right)+\sigma \times CEloss\left(\hat{t},t\right)$$

where θ is the weight of Dice loss and σ is the weights of Cross-Entropy loss. $\hat{t}$ is a segmented mask, and t is the ground truth.

$Diceloss\left(\hat{t},t\right)$ is Dice loss for multi-classes segmentation and this loss is presented as follows:

(11) $$Diceloss\left(\hat{t},t\right)=\frac{1}{P}\times \left(1-\overset{P}{\underset{i}{\sum }}Dicescore({\hat{t}}_{i},\;t_{i}\right))=\frac{1}{P}\times \left(1-\overset{P}{\underset{i}{\sum }}\frac{2{\hat{t}}_{i}t_{i}}{{\hat{t}}_{i}+t_{i}}\right)$$

The weighted Cross-Entropy loss is $CEloss\left(\hat{t},t\right)$, and this is defined as an equation as follows:

(12) $$CEloss\left(\hat{t},t\right)=-\overset{K}{\underset{i=1}{\sum }}\varphi_{i}t_{i}log({\hat{t}}_{i})$$

where $\varphi_{i}$ is the weight of each class and *P* presented the number of classes. We choose θ = 0.9 and σ = 0.1. The values of $\varphi_{i}$ are 0.1, 0.2, 0.2, 0.3, 0.4, 0.4, 0.4 for background, right lung, left lung, spinal cord, heart, esophagus, and trachea, respectively. Our model is implemented using the Pytorch framework. We use Adam optimizer [64] and train our model in 30 epochs. We employ horizontal flip, elastic transform, and rotate augmentations. The model is trained following the K-Fold cross-validation scheme with K = 5. The model is done after 20 h using only GTX 2080Ti—11GB and takes only 20 s for the inference of a patient approximately.

#### 4.3.2. With SegTHOR Dataset

We address the problem as five classes segmentation task. There are four OARs and a background. Because of one pixel for one class, we select the Softmax function to handle this task. Our method is evaluated in the Dice and HD metrics. A combination of Dice loss and Cross-Entropy loss is the same as with the case of the StructSeg dataset. A small difference is that the $\varphi_{i}$ are: 0.2, 0.5, 0.5, 0.5, 0.5 for background, heart, esophagus, trachea, and aorta, respectively. Our model is implemented using the Pytorch framework. We use Adam optimizer [64] and train in our model in 30 epochs. We employ horizontal flip, elastic transform, and rotate augmentations. The model is trained following the K-Fold cross-validation scheme with K = 4. The model is done after 16 h using only GTX 2080Ti-11 GB and takes only 20 s for the inference of a patient approximately.

### 4.4. Performance

#### 4.4.1. With SegTHOR Dataset

**Figure 7:**
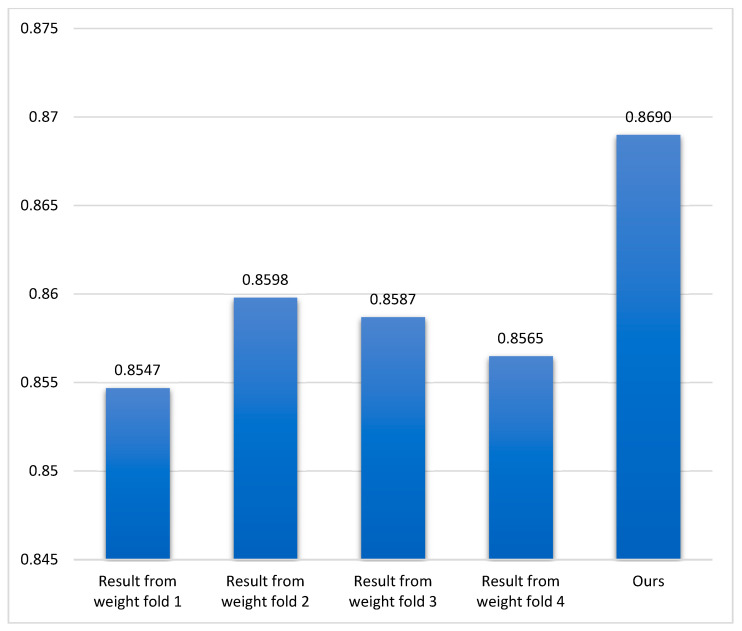
This graph shows the average Dice score value in esophagus segmentation of our method compared to using a separate weight. The performance got from the test set of SegTHOR ISBI 2019 challenge.

In this section, the performance of various methods on the test set is presented. Table 1 shows the Dice score and HD score of esophagus segmentation comparison between our proposed method with others in the test set of the SegTHOR dataset. Our results got the best scores in both Dice and HD metrics. All values are computed by the online website https://competitions.codalab.org/competitions/21145 (accessed 28 May 2021). Figure 7 presents average Dice scores in esophagus segmentation of our method compared to using a separate weight from the test set.

#### 4.4.2. With StructSeg Dataset

**Figure 8:**
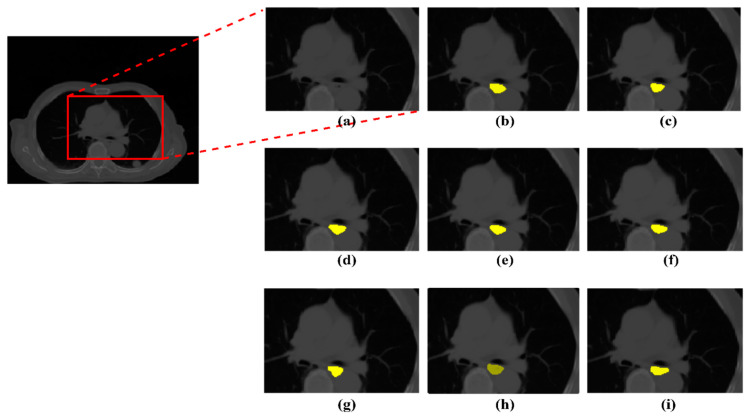
Visualization of esophagus segmentation results from validation set. The yellow area indicates the segmented region. (**a**) Small 2D patch; (**b**) Result of U-Net-cbam-resnet34; (**c**) Result of U-Net-cbam-seresnext50; (**d**) Result of U-Net-scse-resnet34; (**e**) Result of U-Net-scse-seresnext50; (**f**) Result of U-Net-no_att-resnet34; (**g**) Result of U-Net-no_att-se_resnext50; (**h**) Result of our method; (**i**) Ground Truth.

This section presents the performance of various methods on the whole dataset. Table 2 shows the results of K-Fold results on the training set. At first, we experimented with some variant U-Net networks combined with two types of backbone. We select res-net34 and SEResNext50 pre-trained on ImageNet for comparison. Resnet34 is lightweight enough, and SEResNext50 is deep enough for our computing resource. Besides, different attention mechanisms are also involved in evaluating. Secondly, we analyze the efficiency of using the attention mechanism in the decoder. In our experiments, we implemented spatial and channel squeeze and excitation (SCSE) [65] block and integrated it into the decoder block as the same as convolutional block attention module (CBAM) [66] and our spatial attention. Table 2 shows the effectiveness in the Dice metric of our proposed spatial attention mechanism compared to other kinds of attention mechanisms in evaluating the training set. Our approach got Dice, and HD scores in esophagus segmentation outperform different methods. Figure 8 introduces the visualization of esophagus segmentation results from the validation set.

#### 4.4.3. Discussions

While most previous studies evaluate their results on a single data set, we evaluated our method on two datasets to demonstrate the effectiveness of our model. With additional experiments, we tried to evaluate whether the previous methods work for both datasets equally. We selected some methods and applied them to both datasets of StructSeg and SegTHOR. The results in Table 1 and Table 2 show that previous methods are a bit overfit to one dataset, while our approach turned out to be effective for both datasets. These experiments demonstrate that our method is effective on multiple datasets and is not overfit to only one dataset like the other methods. Table 3 shows how results in the test set of the SegTHOR challenge are different between batch normalization (BN) [67] and GN in our model. Note that GN helps our approach to generate results with higher performance in both Dice and HD metrics.

## 5. Conclusions

We proposed a novel U-Net with an attention mechanism combined with using the STAPLE algorithm method as a post-processing step to address esophagus segmentation from chest CT scan challenge. The network leverages a pre-trained model from ImageNet for the encoder part to better extract the CT scan context. By using the spatial attention module, the decoder understands the location of organs better than other attention methods. Our network used 2D images for training to save a massive amount of computing resources compared to 3D volumes. The experiments show the effectiveness and stability of the approach. Our method employed SegTHOR and StructSeg 2019 datasets for evaluation, and the experimental results show that our method achieved promising results in esophagus segmentation. While most previous studies evaluate their approaches on a single data set, we assess our method on two datasets containing the esophagus to demonstrate the effectiveness of our model. The results prove that our method is suitable for esophagus segmentation. Our esophagus segmentation results outperform others in both Dice and HD scores in both SegTHOR and StructSeg 2019 datasets, which presents the stability of our model. The development of the proposed method brings valuable information for physicians and specialist doctors during radiotherapy treatment on the esophagus problem. Although the primary purpose of our method is for esophagus segmentation, we believe that our approach can extend to other organ segmentation with promising results.

## Figures and Tables

**Table 1:** Dice score and HD score of esophagus segmentation comparison between our proposed method with others in the test set of SegTHOR dataset. All values are computed by the online website https://competitions.codalab.org/competitions/21145 (accessed 28 May 2021).

Method	Dice	HD
Lachinov et al. (2019) [40]	0.8303	-
Zhang et al. (2019) [41]	0.7732	1.6774
Chen et al. (2019) [42]	0.8166	0.4914
Vesal et al. (2019) [43]	0.8580	0.3310
Wang et al. (2019) [44]	0.8597	0.2883
He et al. (2020) [45]	0.8594	0.2743
Han et al. (2019) [46]	0.8651	0.2590
U-Net-scse-seresnext50	0.8479	0.3414
U-Net-no_att-resnet34	0.8381	0.3754
U-Net-no_att-se_resnext50	0.8469	0.3652
Ours	0.8690	0.2527

**Table 2:** Dice score and HD95 score of esophagus segmentation comparison between our proposed method with others in StructSeg 2019 training set.

Method	Dice	HD95
MTL-WMCE [45]	0.6055	28.96
U-Net-cbam-resnet34	0.7490	17.68
U-Net-cbam-seresnext50	0.7590	17.97
U-Net-scse-resnet34	0.7606	19.90
U-Net-scse-seresnext50	0.7762	11.31
U-Net-no_att-resnet34	0.7575	12.43
U-Net-no_att-se_resnext50	0.7705	14.39
Ours	0.7784	11.28

**Table 3:** The comparison of Dice score and HD score in esophagus segmentation between using BN and GN in our proposed method. All values are computed from the test set of SegTHOR challenge by online website https://competitions.codalab.org/competitions/21145 (accessed 28 May 2021).

Normalization Technique	Dice	HD
BN	0.8667	0.2748
GN	0.8690	0.2527

## Data Availability

Data was obtained from SegTHOR challenge https://competitions.codalab.org/competitions/21145 (accessed on 28 May 2021).
